# Interpreting Frequency Responses to Dose-Conserved Pulsatile Input Signals in Simple Cell Signaling Motifs

**DOI:** 10.1371/journal.pone.0095613

**Published:** 2014-04-18

**Authors:** Patrick A. Fletcher, Frédérique Clément, Alexandre Vidal, Joel Tabak, Richard Bertram

**Affiliations:** 1 Department of Mathematics, Florida State University, Tallahasee, Florida, United States of America; 2 Project-Team MYCENAE, Inria Paris-Rocquencourt Research Centre, Le Chesnay, France; 3 Laboratoire Analyse et Probabilités EA 2172, Université d'Évry-Val-d'Essonne, Evry, France; 4 Department of Mathematics and Department of Biological Science, Florida State University, Tallahassee, Florida, United States of America; 5 Department of Mathematics and Programs in Neuroscience and Molecular Biophysics, Florida State University, Tallahassee, Florida, United States of America; Georgia State University, United States of America

## Abstract

Many hormones are released in pulsatile patterns. This pattern can be modified, for instance by changing pulse frequency, to encode relevant physiological information. Often other properties of the pulse pattern will also change with frequency. How do signaling pathways of cells targeted by these hormones respond to different input patterns? In this study, we examine how a given dose of hormone can induce different outputs from the target system, depending on how this dose is distributed in time. We use simple mathematical models of feedforward signaling motifs to understand how the properties of the target system give rise to preferences in input pulse pattern. We frame these problems in terms of frequency responses to pulsatile inputs, where the amplitude or duration of the pulses is varied along with frequency to conserve input dose. We find that the form of the nonlinearity in the steady state input-output function of the system predicts the optimal input pattern. It does so by selecting an optimal input signal amplitude. Our results predict the behavior of common signaling motifs such as receptor binding with dimerization, and protein phosphorylation. The findings have implications for experiments aimed at studying the frequency response to pulsatile inputs, as well as for understanding how pulsatile patterns drive biological responses via feedforward signaling pathways.

## Introduction

In endocrine and other systems, oscillations or rhythmic pulses can be more efficient in evoking responses than the same input dose given at a constant level. For instance, calcium oscillations can evoke enhanced gene expression compared to a fixed level in lymphocytes [1]. Pulses of insulin and glucagon are more efficacious at stimulating glucose uptake [2], [3] or production [4], respectively, than an equivalent constant level of hormone. In the stress response system, ultradian pulses of corticosteroids (corticosterone in rodents, cortisol in humans) occur at a frequency of roughly once per hour with a circadian rise and fall in amplitude, and abnormal pulsatility has been linked to depression [5]. During the ovarian cycle, gonadotropin-releasing hormone (GnRH) is secreted in pulses with frequency that varies from once per five hours to once per hour in women [6]. This signal drives gonadotrophs in the pituitary gland to produce the gonadotropins follicle-stimulating hormone (FSH) and luteinizing hormone (LH) preferentially in response to low and high frequency GnRH pulses, respectively. A pulsatile pattern of GnRH within an appropriate frequency range supports reproductive function, while a constant GnRH input at the same mean dose is ineffective [7], [8], [9].

In a target system with an increasing steady-state input-output relationship, pulses of increasing frequency will elicit an increasing response, since increasing pulse frequency alone leads to an increase in input dose. In this situation, it is difficult to distinguish the direct effect of frequency from the effect of input dose. However, an increase in the input frequency is often accompanied by a change in other features of the input. The mean dose may increase with frequency due to a concomitant increase in pulse amplitude or pulse duration, as occurs with synaptic facilitation [10] or with oxytocin pulses during parturition [11], respectively. In other systems, particularly where there are constraints on the production or secretion of a hormone, the mean dose decreases with pulse frequency. For instance in the ewe, as GnRH pulse frequency increases in response to increasing levels of estradiol, there is a striking decrease in pulse amplitude and a modest decrease in pulse duration, leading to a decrease in the average GnRH dose per pulse [12]. It is therefore of interest to understand how systems respond when more than one pulse parameter varies at a time.

Experiments and mathematical modeling studies aimed at understanding the responses to pulsatile inputs often use pulses at increasing frequencies with no change in other pulse characteristics, leading to increases in input dose. An alternative experimental approach sometimes used is the idea of compensating for changes in pulse frequency by adjusting the pulse amplitude to maintain a constant input dose at all stimulation frequencies. For instance, this was used to show that the frequency preference for gonadotropin subunit primary transcript production in gonadotrophs occurs even with a fixed input dose of GnRH [13], [14]. In an experimental context, it is thus desirable to understand the consequences of adjusting pulsatile signals to conserve total dose.

**Figure 1:**
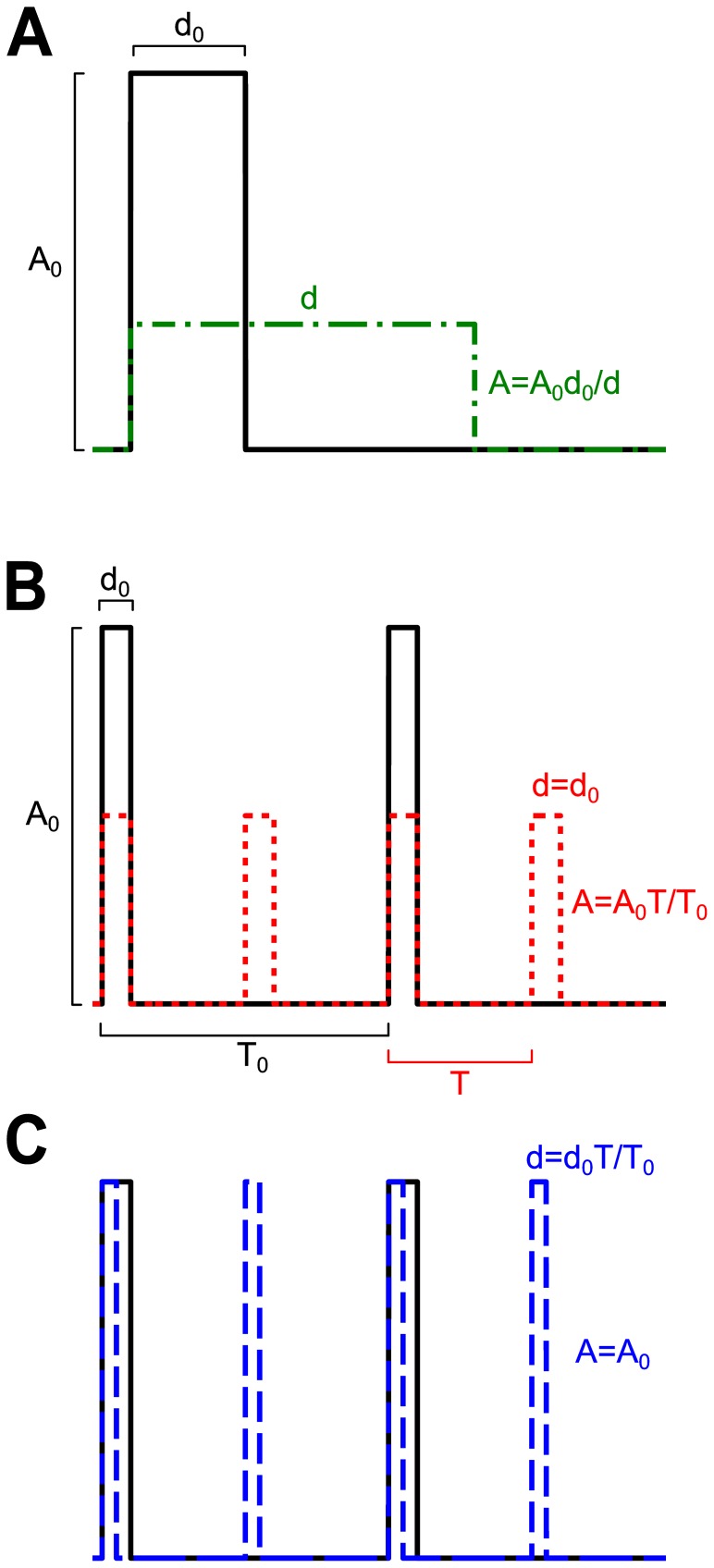
Dose conservation for rectangular pulse signals. (**A**) A pulse that is large and brief (black curve, amplitude A_0_ and duration d_0_) will have the same dose as a small and long pulse (green dash-dotted curve) as long as the new pulse satisfies A = A_0_d_0_/d. (**B**) For periodic signals, the initial signal (black curve) is defined by its amplitude A_0_, duration d_0_, and period T_0_, and its mean dose per period is A_0_d_0_/T_0_. When the pulse period is changed from T_0_ to T, the dose can be conserved by changing the amplitude to A = A_0_T/T_0_ (**B**, dotted red curve) or the duration to d = d_0_T/T_0_ (**C**, dashed blue curve), such that Ad/T = A_0_d_0_/T_0_. We refer to these as amplitude compensation and duration compensation, respectively.

When the dose of input is conserved, what pattern of pulses is best at stimulating responses? The dose of an input signal can be packaged in many ways. For a single pulse, the same dose may be given as a brief large-amplitude pulse, or as a long small-amplitude pulse (Fig. 1A). Alternatively, the input may be broken up into repeated pulses at different frequencies. To ensure that the average dose per pulse is maintained across frequencies, the amplitude or duration of rectangular pulses can be chosen to vary inversely with the pulse frequency (Fig. 1B,C). We refer to these input dose conservation strategies as amplitude compensation (red dotted curve) and duration compensation (blue dashed curve), respectively. Is there a higher output in response to infrequent large pulses, or to frequent small pulses (amplitude compensation)? Is it better to give long pulses with long intervals, or brief pulses with brief intervals (duration compensation)? In general, the answers to these questions depend on the properties of the target system, which can be complex and include negative and positive feedback loops. Here we aim to find rules that describe how to package the input signal for maximal output for the simpler class of single-branch feedforward systems, which are a prevalent building block of intracellular signaling systems.

In this paper we show that some mathematical models for feedforward intracellular signaling motifs, including receptor binding and phosphorylation, exhibit frequency selectivity to pulsatile signals in which the total dose is conserved. We show that this selectivity differs depending on the manner in which the signal is adjusted (amplitude or duration compensated) to maintain constant total dose. We then develop minimal models to analyze the cause of this frequency dependence and show that the relationship between the size of the output and the input frequency can be predicted by the concavity of the input-output function. Finally, we demonstrate that the principles deduced from studying the minimal models hold for the more realistic intracellular signaling motifs.

## Results

### Receptor-ligand binding and dimerization

**Figure 2:**
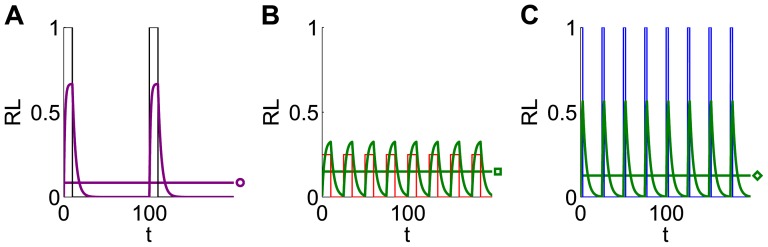
Responses of the ligand-receptor binding model to dose conserved input pulses. (**A**) An initial signal (black) elicits a response in the fraction of bound receptors (RL, purple) with mean value shown by a horizontal line and circle. The initial signal has A_0_ = 1 and d_0_ = 10 at frequency f_0_ = 0.01. (**B**) Responses to input signals with four times the initial frequency are shown in green, with input dose conservation by amplitude compensation (red). (**C**) As in (B), but with duration compensation (blue). The mean response in both (B) and (C) is increased compared to (A).

**Figure 3:**
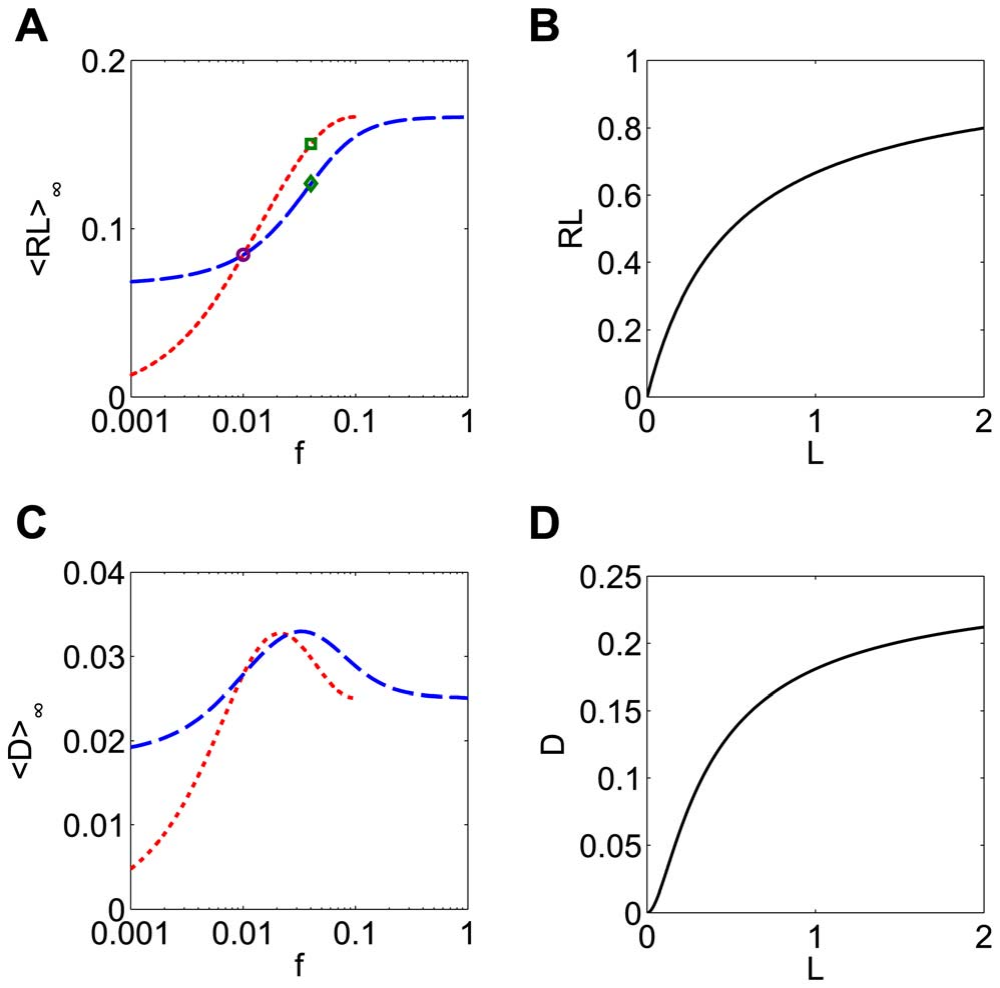
The frequency response differs for systems with different steady state nonlinearity. (**A**) The ligand-receptor binding model exhibits a monotonic-increasing mean concentration of mean bound receptor (<RL>_∞_) to both amplitude and duration compensated signals (red dotted and blue dashed curves, respectively). The circle, square, and diamond indicate the mean response to the signals shown in Figure 2. Note that for amplitude compensated signals, the fixed duration d_0_ = 10 limits the maximum frequency to f = 0.1. (**B**) The steady state fraction of bound receptors (RL) is a monotonic increasing, concave function of the input ligand concentration (L). (**C**) Adding receptor dimerization to the system results in bell-shaped responses of mean level of dimer (<D>_∞_) to both amplitude and duration compensated signals (red and blue curves, respectively). (**D**) The steady state level of dimer (D) is a sigmoidal function of the input ligand concentration. For (A) and (C), A_0_ = 1 and d_0_ = 10 at frequency f_0_ = 0.01 for both amplitude and duration compensated signals.

The first event in any target cell response to a hormone pulse is the binding of the hormone to its receptor. For illustrative purposes, we will first consider a simple generic model of this signaling event. We define *[R]* and *[L]* to be the concentration of unbound receptor and hormone ligand, respectively. Dividing both quantities by the total amount of receptor then gives dimensionless quantities, R and L. Upon binding of the hormone to its receptor, the bound receptor *RL* is formed, as is illustrated by the reaction diagram:
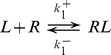
If we consider the total amount of receptor to be conserved (i.e. *L+R = 1*), the fraction of hormone-bound receptor can be described by a single ordinary differential equation (ODE):
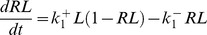
(1)where *RL* is the fraction of ligand-bound receptors, and 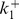 and 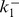 are the binding and dissociation rates, respectively. Without trying to model a specific receptor system, we use 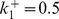 *s^−1^* and 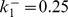 *s^−1^*. During a pulse of ligand with amplitude *A_0_*, duration *d_0_*, and period *T_0_*, the fraction of bound receptor will rise at a rate dependent on the input amplitude, while dissociation occurs between pulses at a constant, slower rate (Fig. 2A). For this and all subsequent figures, the initial signal parameters A_0_, d_0_, and T_0_ are indicated in the figure legend, and the set of input signals used is generated from the appropriate dose conservation protocol described in Figure 1. If the frequency is then increased fourfold, the mean fraction of bound receptor during one input period, *<RL>_∞_*, increases even though the mean input dose is conserved by adjusting pulse amplitude (amplitude compensation, Fig. 2B) or pulse duration (duration compensation, Fig. 2C). This results in a monotonic increasing frequency response (Fig. 3A). The response to amplitude compensated signals (red dotted curve) and duration compensated signals (blue dashed curve) have similar shapes, although amplitude compensated signals elicit a steeper response with a larger dynamic range.

A common next step in many hormone receptor signaling systems is dimerization of the receptor. For example, steroid hormone receptors, such as glucocorticoid, estrogen, and androgen receptors, homodimerize before binding to their DNA target sites [15]. A simple model for this is illustrated by the following reaction diagram:
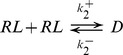
where D represents homodimers of bound receptors. We add terms to the previous ODE and add a second equation for *D* resulting in the system:
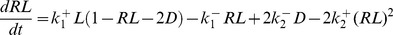
(2)

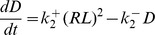
where 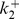 and 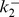 are the association and dissociation rates of the dimer. Here, we use 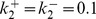 *s^−1^*, with 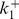 and 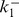 as before. Considering the mean level of dimer, *<D>_∞_*, as the output, we now observe a bell-shaped frequency response for both amplitude and duration compensated signals (red dotted curve and blue dashed curve, respectively, Fig. 3C). Again, the response is steeper and has a larger range when amplitude compensation is used, and the peak response does not happen at the same frequency for duration compensated pulses.

A key distinction between the two models is their steady state nonlinear input-output functions. The receptor-ligand binding model alone exhibits a concave down, monotonic increasing concentration of bound receptor as a function of the input amplitude of ligand (Fig. 3B). Adding receptor dimerization leads to a sigmoidal steady state concentration of dimer as a function of the input ligand concentration (Fig. 3D), even though there is little overall impact on the shape of the curve for *L* away from zero. In the following sections, we will see that the shape of this nonlinearity plays a key role in explaining the shape of the frequency response curve. We will also show that the kinetics of the system help to explain why the peak response is different between the two types of dose conserved signals.

### Nonlinearity selects for input amplitude

We first investigate how a simple nonlinearity determines the magnitude of response in a feedforward system. Consider a signaling molecule, *b*, that is produced at a rate given by a nonlinear production rate function *F* of the input signal *γ(t)*. The ODE governing the concentration of *b* is:
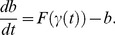
(3)We will refer to this as the b-model. The function *F* could be thought of as a rapid equilibrium approximation to fast signaling dynamics that occur prior to the production of *b*. A similar model was shown to respond to increases in mean input dose in a study of GnRH receptor induced signaling pathways (Model 1 in [16]). The steady state level of *b* in response to a constant input *γ(t) = A* is simply *F(A)*. For a rectangular pulse input, *b* will rise toward the steady state during a pulse, and will decay toward zero after the pulse ends. With periodic input pulses, there is a steady-state periodic response in *b*. We will consider the average value of *b* during one period to be the output of the system. To compute this, we obtain an equation for the time average of *b*, *<b>*, by time averaging equation 3. For periodic rectangular pulse inputs, the asymptotic value of *<b>* from the time averaged ODE is
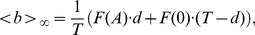
(4)where *A*, *d*, and *T* are the amplitude, duration and period of the input pulses. We chose a time constant τ_b_ = 1 in Eq. 3 for simplicity since it does not affect the steady-state mean value of *b*, only the time it takes to approach this mean value.

Given a particular choice of a nonlinear production rate *F(γ)* and a fixed mean input dose, we can find the pulse shape that will maximize the response by first considering the period *T* to be fixed at a value *T_0_*. To conserve the input dose during the pulse, the pulse amplitude is inversely proportional to the pulse duration (*A = A_0_d_0_/d*, Fig. 1A). We illustrate this for particular choices of monotonic increasing *F*, for which more input (*γ*) leads to more activation of downstream signaling (*b*).

**Figure 4:**
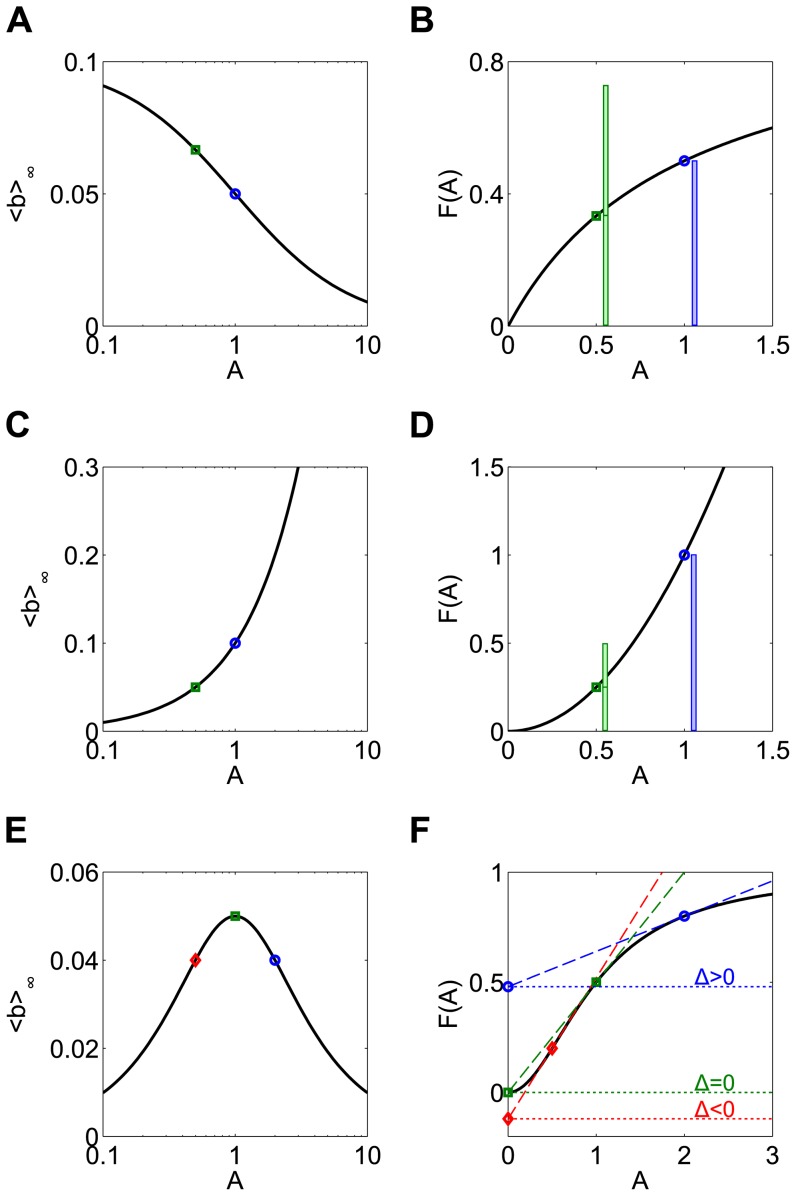
The optimal pulse shape depends on the shape of the nonlinearity. (**A**) The response is maximized for a sublinear production rate function when the pulse amplitude is minimized. A_0_ = 1 and d_0_ = 10 at frequency T_0_ = 100 so the minimum amplitude possible is A = A_0_d_0_/T_0_ = 0.1. (**B**) When the duration of the initial signal (blue circle) is doubled (green square), the amplitude of input is halved. Because of the saturating shape of the production rate function (here F(A) = A/(A+1) is shown), the production of b caused by two small pulses (green bars) is more than the production caused by one large pulse (blue bar), despite the input having the same total dose. (**C**) Superlinear production rate functions lead to large responses for large pulse amplitudes. (**D**) The production of b caused by two small pulses (green bars) is less than the production caused by one large pulse (blue bar). The production rate function is F(A) = A^2^. (**E**) Sigmoidal production rate functions give rise to bell-shaped response. (**F**) For low amplitude input (red diamond), F(A) is superlinear, so the response is increasing. For high amplitude input (blue circle), the production rate function is saturating, so a decreasing response is expected. A maximal response therefore occurs at an intermediate frequency (green square). The slope of the response curve (<b>_∞_) is proportional to Δ, the difference between F(0) and the y-intercept of the line tangent to F(A). The peak frequency occurs when Δ = 0. The production rate function is F(A) = A^2^/(A^2^+1).

When *F* is a sublinear, strictly concave down function (as is the saturating steady state for the receptor model in Fig. 3B), <*b>_∞_* is larger for smaller-amplitude inputs (Fig. 4A). To understand this, recall that if the duration of a pulse is doubled, the pulse amplitude will be halved to conserve dose. This is the same as having two small pulses instead of one large pulse. The production rate of *b* during the small pulse is less than halved due to the saturating shape of the production rate function *F*: 
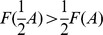
. Thus the longer pulse at half the amplitude will generate a larger production of *b* (Fig. 4B, green bars) than the shorter large pulse (Fig. 4B, blue bar), despite the fact that the input dose was the same.

Results are quite different if *F* has different concavity. If *F* is linear, then <*b>_∞_* is the same for all pulse amplitudes, since 
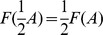
. This invariance of the mean response for dose conserved inputs is not limited to rectangular pulses but occurs with any shape of periodic input. If *F* is a superlinear, strictly convex function *F*, the response <*b>_∞_* increases with input amplitude (Fig. 4C). In this case, it is better to give a brief large pulse than a long small pulse, since 
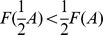
 (Fig. 4D). Finally, if *F* is a sigmoidal function, which is superlinear for small *A* but sublinear for large *A*, a bell shaped <*b>_∞_* response is observed (Fig. 4E). This means the best response will be obtained for an intermediate pulse amplitude. Intuitively, this is expected whenever the set of inputs sample both the superlinear and the sublinear portions of the *F* curve leading to the increasing and decreasing responses, respectively, for the reasons explained above. Perhaps surprisingly, however, the location of the peak response does not occur at the input amplitude corresponding to the inflection point of *F*.

To find the optimal amplitude we compute the slope of the response with respect to *A*, which can be written as:
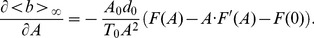
(5)The quantity in parentheses, which we will define as 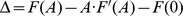, has a geometric interpretation; it is the difference between the y-intercept of the line tangent to *F* at *A*, and *F(0)*. One can therefore predict the shape of the response from shape information contained in *F(A)*, namely its set of tangent lines. Concave down production rate functions have Δ>0 and exhibit larger responses to smaller input amplitudes (Fig. 4F, blue circle). Since Δ<0 for convex functions, one expects larger responses for larger input amplitudes (Fig. 4F, red diamond). For a sigmoidal *F*, the peak of the bell shaped response occurs for the input signal with the amplitude that results in Δ = 0 (Fig. 4F, green square), which is typically not the inflection point of *F*. For the specific sigmoidal curve used here, the Hill function *F(A) = A^2^/(A^2^+K^2^)* with *K = 1*, this occurs when *A = K*.

All the results above apply directly for the case of amplitude compensated signals, since in that case amplitude is a function of pulse frequency (*A = A_0_T/T_0_ = A_0_f_0_/f*, Fig. 1B). Thus, frequency responses to amplitude compensated signals in the b-model occur because of the different input amplitude associated with each frequency. The slope of the frequency response is given by:
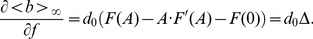
(6)The sign of Δ therefore determines whether the frequency response is increasing, decreasing, or has a critical point.

**Figure 5:**
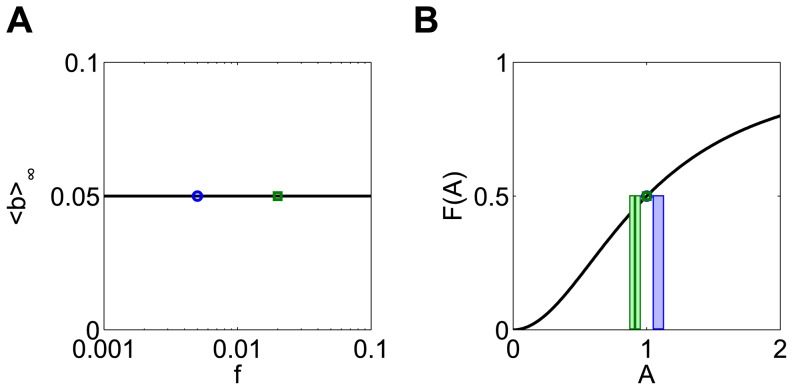
Duration compensated signals yield a flat frequency response in the b-model. (**A**) The flat frequency response. (**B**) Since the amplitude is fixed for all frequencies for duration compensation, doubling the frequency of an initial signal leads to production of ***b*** at the high frequency (green bars) that exactly matches the production caused by one pulse at the low frequency (blue bar). A_0_ = 1, d_0_ = 10, and T_0_ = 100.

Frequency responses do not occur in this model for duration compensated inputs. The response is the same at all frequencies when the dose is conserved using pulse duration, since pulse amplitude *A_0_* is the same at all frequencies. This means that when the frequency of input is doubled, two pulses at the high frequency produce exactly the same production of *b* as one pulse at the low frequency, leading to a flat response (Fig. 5). This shows that if the input amplitude is fixed, long pulses with long intervals produce the same response as short pulses with short intervals.

Taken together, these results suggest that the b-model responds primarily to the amplitude of the input. The optimal amplitude of input can be determined by examining the shape of the nonlinearity in the system. The system will produce a maximal response when this amplitude of input is used, regardless of how the dose is packaged.

### The role of system kinetics

**Figure 6:**
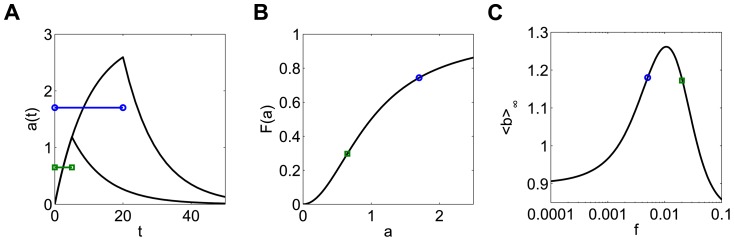
Adding a linear component to the b-model results in frequency sensitivity to duration compensated signals. (**A**) The mean value of *a(t)* during the on and off phases of the pulsatile signal varies with pulse frequency. In particular, the mean value during the on-phase of pulses (colored horizontal lines) decreases as duration decreases, thereby allowing the relevant portions of the nonlinearity of the production rate function F(a) to be sampled by the input signals (**B**). (**C**) For a sigmoidal production rate function, F(a) = a^2^/(a^2^+1), this leads to a bell shaped response. A_0_ = 3 and d_0_ = 10 at frequency f_0_ = 0.01.

The kinetics of chemical reactions that comprise intracellular signaling pathways may affect the response by filtering the input signal. In the b-model, the nonlinear function *F* can be thought of as the steady state input-output relation of the signaling system, so in that case the system kinetics were not accounted for. We therefore extend the b-model to incorporate kinetics by introducing an intermediate variable, *a*, where the production of *a* is linear in the input signal, *γ(t)*. The model equations are:
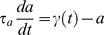
(8a)

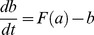
(8b)where *τ_a_* is the time constant of *a*, and *τ_b_* is again 1 for simplicity. Unless otherwise stated, *τ_a_* = 10 for the following results. We will refer to this as the ab-model. The first stage in the cascade acts as a linear low-pass filter on the amplitude of the input, with *τ_a_* controlling the cutoff frequency. This means the variable *a* achieves larger values during long pulses than it does during short pulses, effectively converting pulse duration into amplitude before the nonlinear stage (Fig. 6A). Here, we treat the case of small pulse duration relative to the period. Considering the mean value of *a(t)* during a pulse, *<a>_on,∞_*, as an approximation (green squares and blue circles, Fig. 6) we see now that *<a>_on,∞_* varies enough to sample the relevant parts of the nonlinear function *F* and give rise to a frequency response (Fig. 6B). A bell-shaped frequency response to duration compensated signals (Fig. 6C) is demonstrated for a sigmoidal *F* function.

**Figure 7:**
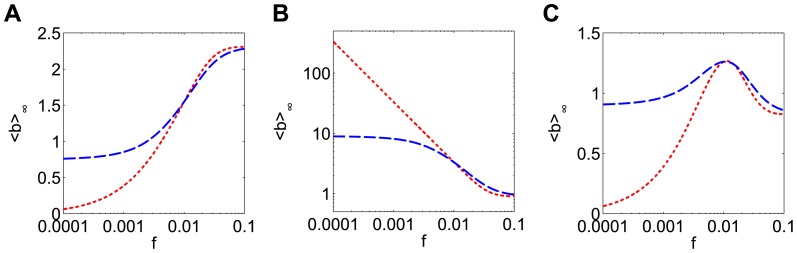
The ab-model displays the responses predicted by the nonlinear production rate function. The model has increasing (**A**), decreasing (**B**) and bell-shaped (**C**) responses when the production rate function is sublinear, superlinear, and sigmoidal, respectively. Frequency responses for amplitude compensated signals and duration compensated signals are shown in red (dotted curve) and blue (dashed curve), respectively. A_0_ = 3 and d_0_ = 10 at frequency f_0_ = 0.01.

Using the same three examples of sublinear (Fig. 7A), superlinear (Fig. 7B), and sigmoidal functions *F* (Fig. 7C), the ab-model exhibits responses similar to the amplitude-compensated case of the b-model when either amplitude compensated signals (red dotted curves) or duration compensated signals (blue dashed curves) are used as inputs.

As seen with the receptor-ligand binding and dimerization model, the amplitude compensated inputs elicit steeper frequency responses with a larger range of mean outputs than duration compensated inputs. These differences can be explained by the filtering effect of the kinetics of the linear stage of the cascade. The range of response is smaller for duration compensated signals since the linear filter leads to significant changes in *<a>_on,∞_* only for an intermediate range of frequencies near the cutoff frequency. At low frequencies, pulses have a large duration and the value of the variable *a* rises to its equilibrium value, *a = A_0_*, long before the end of the pulse. The input to *b* therefore consists of rectangular pulses whose amplitude changes very little with frequency and flat frequency response is observed regardless of the choice of *F*. At high frequencies, the amplitude of oscillations in *a* gets very small, so the input appears to be a constant mean level and the response is again flat.

**Figure 8:**
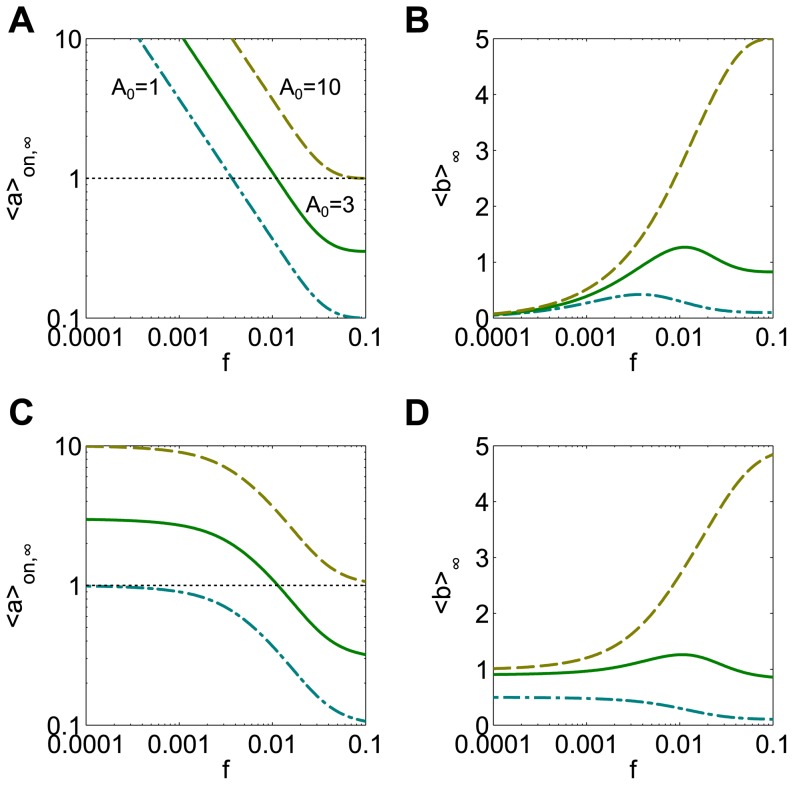
Input signal dose can affect whether a bell-shaped response is observed. The production rate function is F(a) = a^2^/(a^2^+K^2^) with K = 1. (**A**) Amplitude compensated signals with A_0_ = 10 (tan dashed curve) leads to <a>_on,∞_ that is larger than K (i.e. above the horizontal dotted line) for all possible frequencies, while any choice of A_0_ smaller than 10 allows <a>_on,∞_ to cross K (teal dash-dotted curves and green solid curves). (**B**) When A_0_ = 10, all values of <a>_on,∞_ are in the sublinear, saturating part of the sigmoidal F, and a strictly increasing response in <b>_∞_ is observed. When the mean input dose is lower, bell shaped responses are observed, with the peak frequency corresponding to the frequency at which <a>_on,∞_ = K. (**C**) Duration compensated signals may lead to responses of <a>_on,∞_ that are strictly below K, cross K at some frequency, or are strictly above K (teal dash-dotted curves, green solid curves, and tan dashed curves, respectively). (**D**) The frequency responses of <b>_∞_ are strictly decreasing (teal), bell-shaped (green), and strictly increasing (tan), respectively. For each A_0_ used, d_0_ = 10 at frequency f_0_ = 0.01.

In contrast, with amplitude compensated signals the input pulse amplitude varies widely across frequencies and filtering plays a less significant role. The steeper response to amplitude compensated signals can be explained by the observation that the frequency response in *<a>_on,∞_* is always steeper for amplitude compensation (compare green solid curves, Fig. 8A and C).

### Matching input signals to the nonlinear production rate

We now show how the input signal can be matched (or mismatched) to the system's kinetics and nonlinearity and how this changes the frequency response, using the sigmoidal production rate function *F(a) = a^2^/(a^2^+K^2^)* with *K = 1* as an example. Input signals that result in *<a>_on,∞_ = 1* correspond to a peak in the response *<b>_∞_* for both amplitude and duration compensated signals (green curves, Fig. 8). As long as the input signals generate a range of *<a>_on,∞_* values that includes *<a>_on,∞_ = 1*, we will observe a bell-shaped response in the frequency range tested.

When the mean dose of the input signals is changed, demonstrated here by changing the amplitude of the initial signal *A_0_*, the system may no longer display a bell-shaped response. With amplitude compensation, if the mean input dose is sufficiently large, there will be no input frequency that results in *<a>_on,∞_ = 1* (tan dashed curve, Fig. 8A). In this case, all inputs will have large amplitude and therefore will sample only the saturating part of the production rate function *F*, leading to a monotonically increasing frequency response (tan dashed curve, Fig. 8B). For smaller input doses, a bell-shaped frequency response is guaranteed since *<a>_on,∞_* will cross *K = 1* at some frequency, thereby sampling both the sublinear and superlinear portions of *F* (teal dashed-dotted curve and green solid curve, Fig. 8A). As the input dose is decreased, the peak frequency shifts to the left and the amplitude of the response decreases (Fig. 8B). If the input dose is sufficiently small, *<a>_on,∞_* may not cross *K = 1* in the frequency range tested, which would result in the observation of a monotonic decreasing frequency response (not shown).

With duration compensated inputs, there is an intermediate range of input doses where a bell-shaped frequency response is produced (green curves, Fig. 8C,D). For input amplitudes that are too large, the response is again monotonically increasing, since *<a>_on,∞_*>*1* (tan dashed curves, Fig. 8C,D). Alternatively, if the input amplitudes are too small, *<a>_on,∞_*<*1*; this means only the superlinear region of the sigmoidal *F* function is sampled, and there is a monotonically decreasing frequency response (teal dashed-dotted curves, Fig. 8C,D). A bell-shaped response occurs for a more limited range of *A_0_* due to the fact that significant changes in *<a>_on,∞_* only occur for a limited range of frequencies.

**Figure 9:**
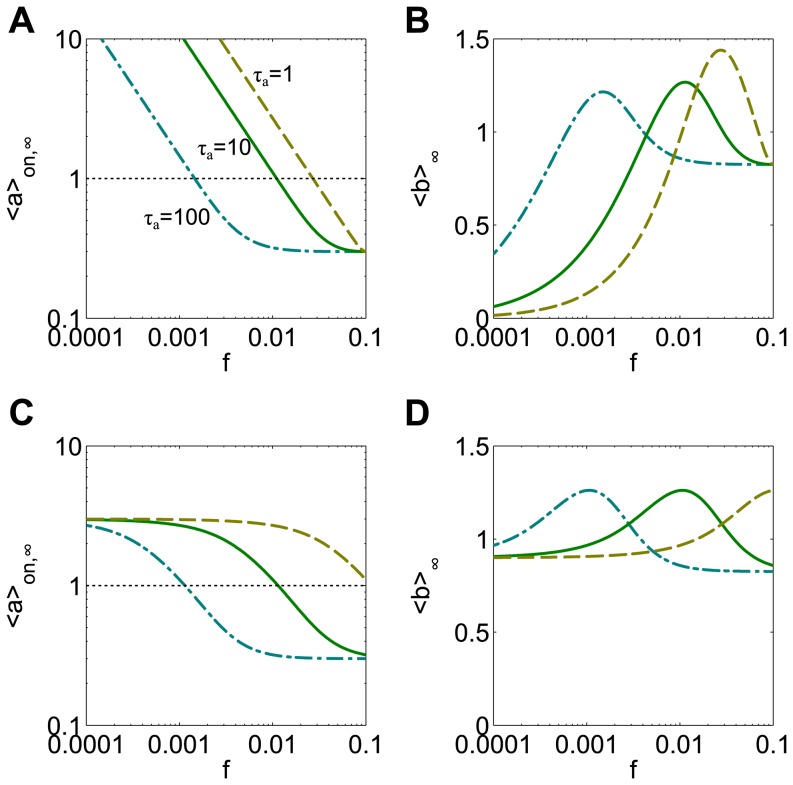
The kinetics of the linear component affect the peak frequency of a bell shaped response. The production rate function is F(a) = a^2^/(a^2^+K^2^) with K = 1. (**A**) Amplitude compensation: when the time constant of variable ***a*** is increased to τ_a_ = 100 (teal dash-dotted curve) or decreased to τ_a_ = 1 (tan dashed curve) from the default value of τ_a_ = 10, the frequency at which <a>_on,∞_ crosses K = 1 shifts to the left or right, respectively. (**B**) The corresponding bell-shaped responses have a peak that shifts to the right accordingly. (**C**) Duration compensation: increasing τ_a_ shifts the response in <a>_on,∞_ and its K crossing point to the right. The fastest system (tan dashed curve) no longer crosses K in the frequency range examined. (**D**) The bell-shaped response shifts to the right accordingly, and <b>_b_ is a strictly increasing response in the frequency range examined. A_0_ = 3 and d_0_ = 10 at frequency f_0_ = 0.01.

From these illustrations we see that even with a sigmoidal production rate function *F*, the frequency response could be increasing, decreasing, or bell-shaped, depending on the magnitude of the input. Thus, the frequency response can be tuned by the strength of the input. Another way that the frequency response can be tuned is through the kinetics of the system. To see this we vary *τ_a_* from its default value of 10 and observe the responses to inputs known to produce a bell-shaped response in a given frequency range (green solid curves, Fig. 9). In this case, the kinetics of the linear filter determine the location of the peak frequency, again by setting the frequency at which *<a>_on,∞_ = 1*.

For amplitude compensated signals faster kinetics (decreasing *τ_a_*) right-shifts the peak frequency, but the range of *<a>_on,∞_* values always includes values above and below *K = 1*. Thus, the response in the limit *τ_a_→0* remains bell-shaped (tan dashed curves, Fig. 9A, B). This corresponds to the response expected from the simpler b-model. When *τ_a_* is increased, the first stage of the cascade responds to inputs more slowly and the filter's cutoff frequency decreases. This leads to a crossing of *<a>_on,∞_* = *1* at a lower frequency and a left-shifted peak response in *<b>_∞_* (teal dash-dotted curves, Fig. 9A, B). If the system kinetics are slow enough, it is possible that a purely decreasing response could be observed, since only values of *<a>_on,∞_<1* would be produced for the tested frequency range (not shown).

When duration compensated signals are used as inputs, the response in *<b>_∞_* is again shifted left or right if *τ_a_* is increased or decreased, respectively (teal dash-dotted and tan dashed curves, Fig. 9C,D). The shift in peak response frequency appears to change linearly with *τ_a_*. Contrary to the case of amplitude compensation, the shape of the response here is unchanged when it is shifted by changes in *τ_a_*. If the system has fast enough kinetics, a purely increasing response may be observed since values of *<a>_on,∞_* attained will all be larger than *K*. In the limit *τ_a_→0*, the response approaches the flat response seen in the b-model. Similarly, if the system has very slow kinetics, the response observed may be decreasing or flat, due to only values smaller than *K* being attained by *<a>_on,∞_* in the tested frequency range.

### Protein phosphorylation: another sigmoidal nonlinearity

We now return to a more realistic model of an intracellular signaling motif. A common signaling motif, ubiquitous in eukaryotic cells, is the reversible covalent modification of a protein. An example of this is the reversible phosphorylation of a protein by kinase and phosphatase enzymes. A well-studied model for such a chemical reaction is due to Goldbeter and Koshland [17], which involves a loop of two coupled enzymatic reactions. Using their original notation we let *W* and W^*^ represent the protein in its dephosphorylated and phosphorylated states, and *E_1_* and *E_2_* represent the kinase and phosphatase, respectively. For the phosphorylation reaction, *E_1_* reversibly binds the unmodified protein *W* to form a complex *C_1_*. The kinase may then phosphorylate the protein, producing W^*^. The dephosphorylation reaction, involving W^*^, *E_2_*, and the complex between them, *C2*, is similar. The reactions involved are:
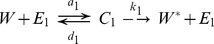


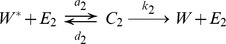
where constants *a_1_*, *a_2_* and *d_1_*, *d_2_* are the complex association and dissociation rates, while *k_1_* and *k_2_* represent the phosphorylation and dephosphorylation rates, respectively. If the total amount of protein, kinase, and phosphatase are each conserved, the system of six chemical species can be described using three ODEs along with three conservation laws:
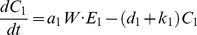


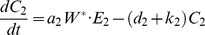


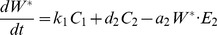
(9)

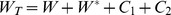


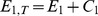


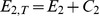
where *W_T_*, *E_1,T_*, and *E_2,T_* are the total protein, kinase, and phosphatase, respectively. Following the binding of a hormone to its receptor, the total available amount of active kinase often increases (for instance, GnRH binding to its receptor leads to increases in protein kinase A activity [18], [19], protein kinase C and mitogen-activated protein kinases – see [20], [21] for review). Therefore, we consider the total kinase *E_1,T_* as the input to the system, and we will consider the initial input signal to be pulses of *E_1,T_* with amplitude *A_0_*, duration *d_0_*, and period *T_0_*. The phosphorylated form of the protein is often the active form so we will consider the mean phosphorylated fraction W^*^, <W^*^>_∞_, to be the relevant output. We normalize by dividing by *W_T_* to show the fraction of phosphorylated protein.

**Figure 10:**
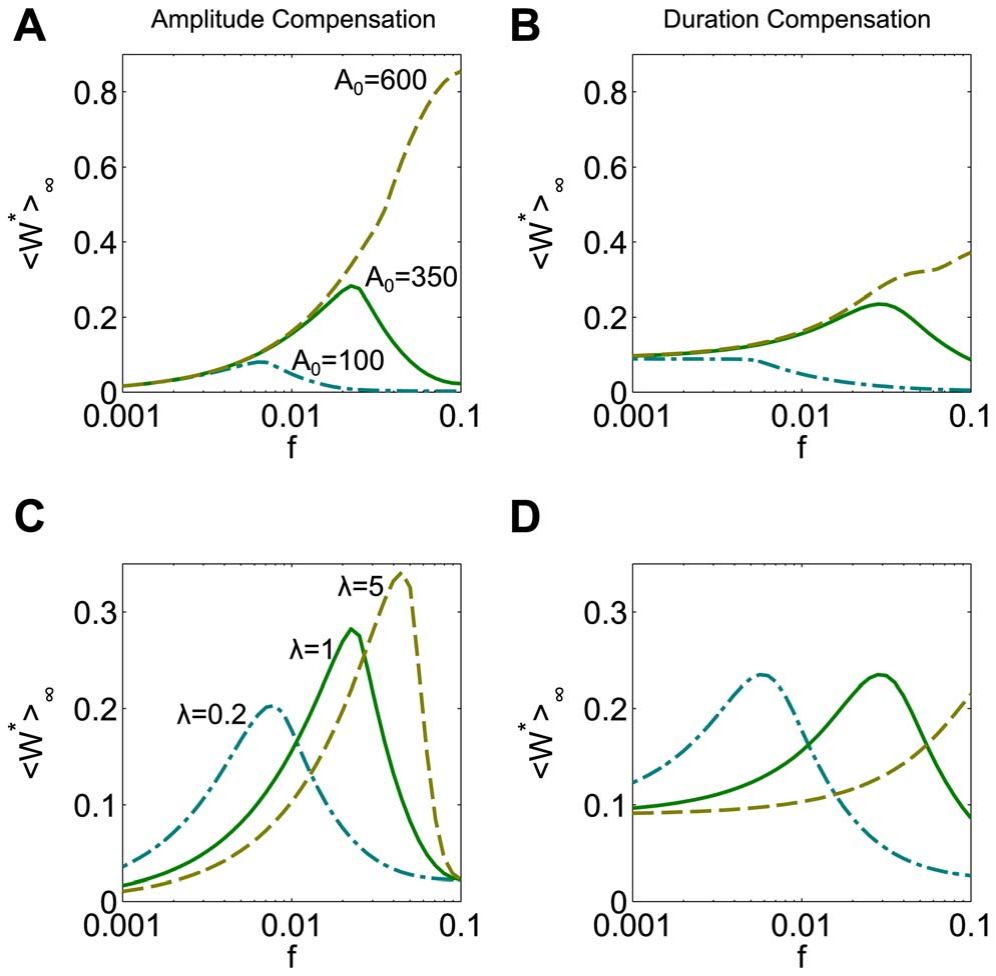
Matching input signal characteristics to the sigmoidal nonlinearity and system kinetics in the Goldbeter-Koshland model. (**A**) Amplitude compensated signals with a sufficiently large A_0_ lead to a monotonic increasing response (tan dashed curve), while signals with smaller A_0_ elicit bell-shaped responses (green solid curve, teal dash-dotted curve). (**B**) Duration compensated signals with the same A_0_ as in (A) elicit monotonic increasing (tan, A_0_ = 600), bell-shaped (green, A_0_ = 350), or monotonic decreasing (teal, A_0_ = 100) responses. For each A_0_ used, d_0_ = 10 at frequency f_0_ = 0.01. (**C**) Multiplying all rate constants by a factor λ shifts the location of the peak response to amplitude compensated input signals (A_0_ = 350) relative to the default system (green solid curve, λ = 1). Slower kinetics lead to a left-shifted response curve, while faster kinetics lead to a right shifted curve with a peak that remains in the frequency range tested. (**D**) For duration compensated signals, a system with slower kinetics (teal dash-dotted curve, λ = 0.2) has a left shifted peak, while fast kinetics (tan dashed curve, λ = 5) may shift the peak beyond the frequency tested. Note that the <W^*^>_∞_ values have been normalized by dividing by *W_T_*. A_0_ = 350 and d_0_ = 10 at frequency f_0_ = 0.01.

At steady state, the fraction of phosphorylated protein is an increasing nonlinear function of the input level of kinase, *E_1,T_*. In the regime where *W_T_*≫*E_1,T_*, *E_2,T_* the steady state concentration W^*^ is a steep sigmoidal function of the input *E_1,T_*, with the half-maximum value set by *E_2,T_* (not shown). To achieve this, we use the parameter values *W_T_ = 1000 nM*, *E_2,T_* = 50 *nM*, *a_1_* = *a_2_ = 50 nM^−1^ s^−1^*, *d_1_* = *d_2_ = 499 s^−1^*, and *k_1_* = *k_2_ = 1 s^−1^*. In this regime, the system exhibits a steep bell-shaped frequency response to dose conserved inputs (Fig. 10, solid green curves). That is, there is an optimal pattern of input pulses that yields a maximal mean level of phosphorylated protein, W^*^. Again, the range of response to amplitude compensated signals is greater and the responses are steeper when compared to duration compensation.

Considering signals with different *A_0_*, we observe qualitatively similar results to the simple cascade model studied in the previous section. For large *A_0_*, the response to both amplitude and duration compensated signals is monotonically increasing, due to the saturating property of the nonlinearity (tan dashed curves, Fig. 10A and B, respectively). For small *A_0_*, the bell-shaped response is retained for amplitude compensated signals (teal dash-dotted curve, Fig. 10A), while it becomes a decreasing response for duration compensated signals (teal dash-dotted curve, Fig. 10B). This is in agreement with the results of the ab-model (Fig. 8B, D).

To study the effect of system kinetics, we multiply all rate constants in the system by a common factor, *λ*. We consider a set of input signals that elicits a bell-shaped response in the system with default parameter values (*λ = 1*, green solid curves, Fig. 10C, D). As was seen in the ab-model (Fig. 9B, D), a system with slower kinetics has a left-shifted peak frequency (*λ = 0.2*, teal dash-dotted curves), while a faster system has a right-shifted peak (*λ = 5*, tan dashed curves) for both amplitude and duration compensated signals (Fig. 10 C and D, respectively). When the system has fast kinetics, the bell-shaped response is retained for amplitude compensated signals (tan dashed curve, Fig. 10C), while the bell-shape may be shifted out of the tested frequency range when duration compensated signals are used (tan dashed curve, Fig. 10D). This means that if the system has fast kinetics, one might observe a flat response when using duration compensated signals, as was observed with the ab-model.

## Discussion

In this study, we sought to understand how simple feedforward signaling pathways best respond to pulsatile inputs, given a fixed total amount of input to the system. We asked whether the system responds better to low-frequency periodic application of brief pulses with large amplitude, or to a high frequency application with small amplitude. A similar question is whether it is better to use long pulses separated by long intervals, or short pulses with short intervals. We framed these questions in terms of finding the maximum in the frequency response to amplitude or duration compensated signals, and found that for simple feedforward signaling motifs the shape of the steady-state input/output function selected for an input amplitude that maximized the response. The kinetics of the system and the mean input dose could also affect the result, largely by reshaping the input signal amplitudes. The results from the study of our minimal ab-model were a good predictor for the responses in the more realistic models of feedforward signaling motifs, namely receptor dimerization and phosphorylation.

There are many ways to conserve the input dose of a pulsatile signal while varying the pulse frequency. The two simplest dose conservation methods, compensating by altering pulse amplitude or pulse duration, produced different frequency responses, and are therefore not equivalent despite having the same input dose. Our finding that the nonlinearity in the input/ouput function selects for an optimal amplitude demonstrates that these responses are not direct responses to frequency, but instead are reflections of the association of an input amplitude to each frequency. Thus, the large dynamic range in the responses to amplitude compensated signals is due to the fact that amplitude compensated signals introduce large variations in signal amplitude in order to conserve dose.

Duration compensation stretches the input signal without introducing variations in input amplitude seen in amplitude compensation. Frequency responses to duration compensated signals in feedforward systems therefore required the conversion of input durations to amplitudes, achieved in the ab-model by using a simple linear low-pass filter. In more realistic systems, this filtering of inputs would be due to the system's kinetics.

Analysis of the ab-model provides some insight into different situations that might affect whether a frequency response is observed when using dose-conserved pulsatile inputs to study cell responses, in particular when feedforward pathways are involved. Our observation that the type of frequency response can change with the total input dose suggests that in laboratory studies, one should determine the frequency response at several values of the total dose. This may be necessary to see the full response characteristics.

A second consideration is the filtering properties due to the system kinetics. We observed that the low-pass filtering of the system modulated the input amplitude, and thus affected the frequency response (Fig. 9). In experiments, the observation of a flat, decreasing, or increasing response could be due to the fact that the range of frequencies tested was not appropriately matched to the system kinetics. Note also that linear dispersion that may occur during transport of pulses of hormone through a perfusion system or blood vessels could introduce low-pass filtering of the input signal. As we observed in the ab-model, the low-pass filtering properties may shift the observed frequency response in the target cell.

The results described in this paper were deduced specifically for single branch feed forward motifs. However, it is common that a ligand may stimulate more than one feedforward branch of signaling pathways, which then converge to a common downstream output. Examples include GnRH-stimulated parallel extracellular signal-regulated kinase (ERK) and nuclear factor of activated T cells (NFAT) pathways which converge to stimulate gonadotropin synthesis [22], or the parallel activation and inhibition seen in *Dictyostelium discoideum* response to cAMP [23], and in the control of the inflammatory response by Toll-like receptor 4 (TLR4) signaling [24]. In these systems, the overall depends on the mechanism by which they converge at the common output. Our results apply to understanding the behavior of isolated signaling motifs, and how the behavior of these motifs link together in longer chains or parallel chains to yield an overall output response is a topic of future research. Another common motif in signaling pathways are feedback loops, where a downstream component of the pathway affects an upstream component. Examples include negative feedback of mitogen-activated protein kinase (MAPK) phosphatases in the GnRH-stimulated ERK signaling pathway [25] and the negative and positive feedback in the epidermal growth factor (EGF) and nerve growth factor (NGF) pathways [24]. The behaviors of systems that include feedback loops were also not considered here.

The variations in GnRH pulse amplitude with pulse frequency reported in the ewe in response to increasing levels of estradiol [12] are similar to the amplitude compensated input signals studied here. Thus, the results demonstrated in this study may help in understanding responses in feed-forward signaling pathways triggered by GnRH. While there have been many detailed experimental (for review, see [20], [21]) and theoretical (for instance, [16], [22], [25]) studies of the GnRH receptor-induced signaling network, it remains to be determined precisely which components are responsible for the bell-shaped frequency responses of LH and FSH production in pituitary gonadotrophs.

In physiological situations, constraints on the dose of the input signal could arise due to high costs of production and secretion of hormone from the signal generating cells. Biological systems may therefore change the pattern of the signal as a way to transmit information. Taken together, our results suggest some mechanisms for how a biological system could adjust the responses in various target tissues, without needing to dramatically change the dose of the signal. If the nonlinear input-output function is different for different target systems, they will respond best to different input amplitudes. Also, the kinetics of different pathways may favor higher or lower frequency pulses. This may help to explain how in some cases, given that a receptor can activate many downstream pathways, some signaling pathways may be optimally stimulated by a specific pattern of input, while others are not.

## Methods

Analytical solutions to periodic rectangular pulse forcing were computed for *RL(t)* in the receptor binding model (Fig. 2) and for *a(t)* in the ab-model (Figs. 6A). The mean output for all models is defined as the time average of the output variable (e.g. *RL(t)*). Due to the periodic input and the globally stable nature of feed-forward systems, the output variable approaches a stable steady-state periodic solution. Thus, we consider the mean output to be the mean over one period *T* of the steady-state periodic solution *x(t)*:
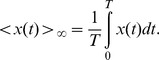
When the analytical solution was available, we computed the mean output at each frequency using adaptive Lobatto quadrature, quadl in MatLab (R2010a, The Mathworks, Natick, MA), with tolerance 1e-6 (Fig. 3A for receptor binding, and Figs. 6–9 for the ab-model, respectively).

The results for the dimerization model and the phosphorylation model (Figs. 3C, 10) were obtained by numerical integration using the ode15s routine in MatLab. Successive on and off phases of the pulsatile input were sequentially simulated. The steady state mean value was approximated by discarding the first portion of the simulation (initial transient behavior) so the system was close to the periodic solution. The mean was computed using ode15s by adding an auxiliary equation, *dx/dt = x*, to the system, where *x* = *D* or x = W^*^ for the dimer and phosphorylation models, respectively. The MatLab code is available at www.math.fsu.edu./~bertram/software/pituitary.
